# Mathematical modeling at the livestock-wildlife interface: scoping review of drivers of disease transmission between species

**DOI:** 10.3389/fvets.2023.1225446

**Published:** 2023-09-06

**Authors:** Brandon H. Hayes, Timothée Vergne, Mathieu Andraud, Nicolas Rose

**Affiliations:** ^1^IHAP, Université de Toulouse, INRAE, ENVT, Toulouse, France; ^2^Ploufragan-Plouzané-Niort Laboratory, The French Agency for Food, Agriculture and the Environment (ANSES), Ploufragan, France

**Keywords:** mechanistic, epizootic, transmission, review, interface, domestic, livestock, wildlife

## Abstract

Modeling of infectious diseases at the livestock-wildlife interface is a unique subset of mathematical modeling with many innate challenges. To ascertain the characteristics of the models used in these scenarios, a scoping review of the scientific literature was conducted. Fifty-six studies qualified for inclusion. Only 14 diseases at this interface have benefited from the utility of mathematical modeling, despite a far greater number of shared diseases. The most represented species combinations were cattle and badgers (for bovine tuberculosis, 14), and pigs and wild boar [for African (8) and classical (3) swine fever, and foot-and-mouth and disease (1)]. Assessing control strategies was the overwhelming primary research objective (27), with most studies examining control strategies applied to wildlife hosts and the effect on domestic hosts (10) or both wild and domestic hosts (5). In spatially-explicit models, while livestock species can often be represented through explicit and identifiable location data (such as farm, herd, or pasture locations), wildlife locations are often inferred using habitat suitability as a proxy. Though there are innate assumptions that may not be fully accurate when using habitat suitability to represent wildlife presence, especially for wildlife the parsimony principle plays a large role in modeling diseases at this interface, where parameters are difficult to document or require a high level of data for inference. Explaining observed transmission dynamics was another common model objective, though the relative contribution of involved species to epizootic propagation was only ascertained in a few models. More direct evidence of disease spill-over, as can be obtained through genomic approaches based on pathogen sequences, could be a useful complement to further inform such modeling. As computational and programmatic capabilities advance, the resolution of the models and data used in these models will likely be able to increase as well, with a potential goal being the linking of modern complex ecological models with the depth of dynamics responsible for pathogen transmission. Controlling diseases at this interface is a critical step toward improving both livestock and wildlife health, and mechanistic models are becoming increasingly used to explore the strategies needed to confront these diseases.

## Introduction

Modeling of infectious diseases at the domestic-wildlife interface is a unique niche within mathematical modeling. Requiring cross-disciplinary competence in infectious disease epidemiology, domestic animal health and livestock production, and wildlife ecology, these models seek to unravel the complex mechanisms behind both disease transmission between ecosystems and disease emergence in novel ecosystems. Developing models at this interface carries its own unique set of challenges. Indeed, entire articles have been written on the subject (1, 2). Simply estimating transmission between species is a burdensome task. There exists difficulty even in defining what constitutes an epidemiologically-relevant contact, as laboratory-based forced contact is different than that experienced under natural circumstances, and observing natural contacts to infer model parameters is a challenging ecological task (1). Further, spillover events are rarely observed but their frequency must be indirectly inferred, so as to inform the means of disease transmission in the non-reservoir population (2).

Transmission drivers for a wide range of pathogens have been well studied among human and domestic animal populations, for which specific epidemiological studies were set-up. In contrast, the transmission dynamics of infectious agents among wildlife species is more difficult to assess (3, 4). Wildlife characteristics ranging from descriptions of movement patterns and contact networks to simply quantifications of host population size are less certain (3, 5–8). The difficulty of observing wildlife species further affects the ability to obtain accurate measurements of disease frequency—and even simply of host population distribution—due to biases among sampled and non-sampled subsets of wildlife populations (3, 9). These uncertainties inherently affect the ability to quantify the transmission potential of a disease among its host population, and these uncertainties must be recognized and accounted for when developing mechanistic models for infectious agents in the context of wildlife populations.

Modeling disease transmission at the interface between domestic and wildlife species, therefore, is a complex equation system involving multiple distinct host and pathogen factors. Within these mathematical models, the modeling frameworks used to represent the transmission dynamics in such context need to account for the specificities of both host populations. However, models also need to remain parsimonious in terms of parameterization, not to add unnecessary uncertainty into the system. Therefore, a balance between model complexity and host population representation needs to be found to capture the transmission dynamics in regard to the available data. This review aims to examine the means of representation of livestock and wildlife species, drivers and mechanisms of transmission in the models, and the main challenges that are yet to be overcome in this field.

## Materials and methods

The literature search was conducted *via* the PubMed and Web of Science databases on 28 February 2023 and performed in accordance with PRISMA guidelines (10). Constructed to capture all articles of mechanistic modeling that accounted for transmission between major livestock species and wildlife, the search—within keywords, title, and abstract—was comprised of the following query: (*livestock* OR *cattle* OR *cow* OR *ruminant* OR *bovine* OR *swine* OR *pig* OR *porcine* OR *sheep* OR ovine OR *goat* OR *caprine*) AND (wild* OR *“wild boar”* OR *buffalo* OR *bison* OR *deer* OR *elk* OR *ibex* OR *badger*) AND *transmission* AND (*simulation* OR math* OR *stochastic* OR *estimation* OR *inference*) AND *model*. The search was restricted to mammalian species, as the ecological processes behind the drivers of transmission of non-mammalian epizootic diseases of major concern, notably highly-pathogenic avian influenza, were considered too distinct and deserving of their own independent review. No date limitation was specified, and the English language was indirectly specified through search terminology.

**Figure 1:**
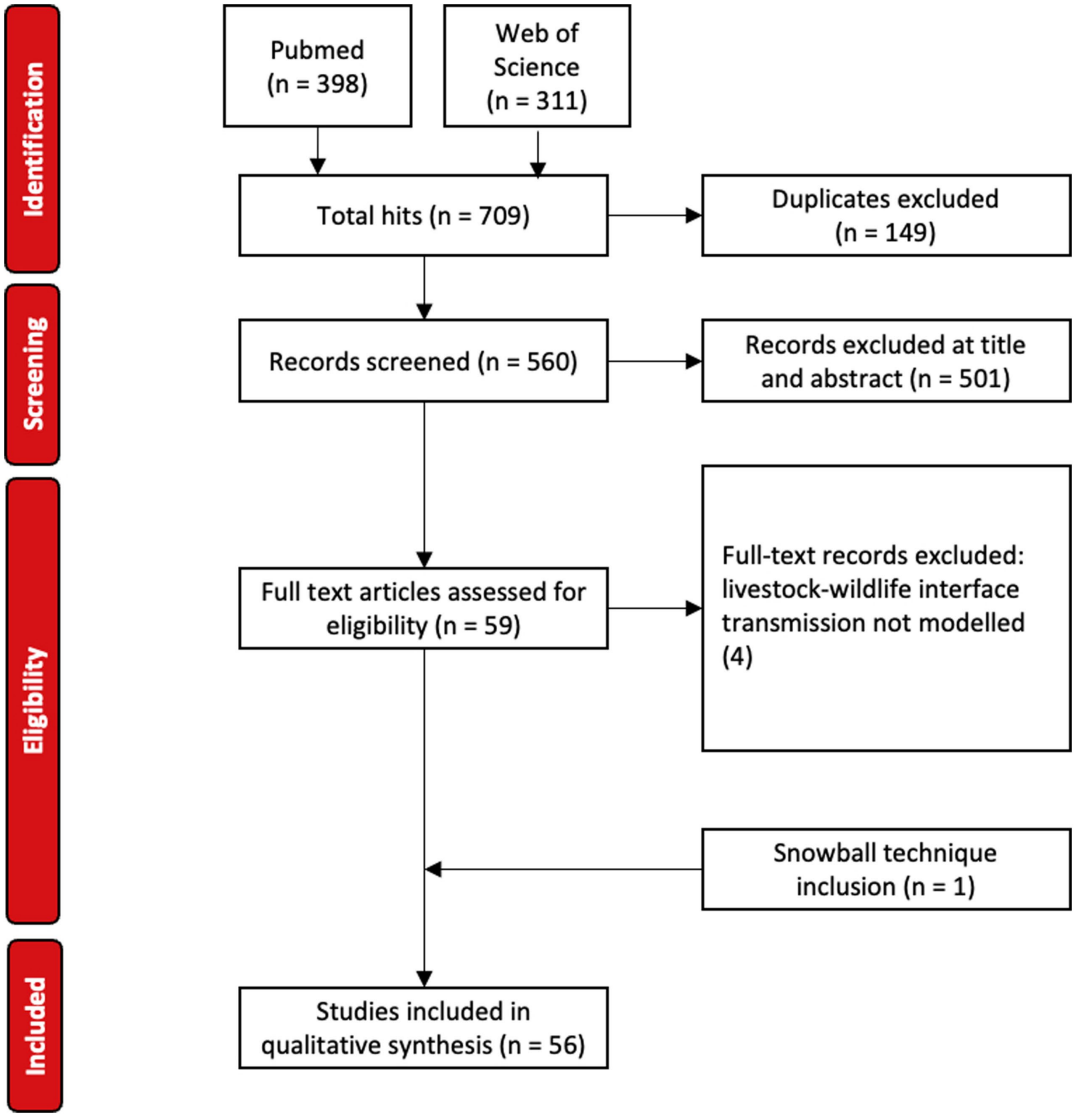
PRISMA flow diagram for article selection.

A total of 709 articles were retrieved (PubMed 398, Web of Sciences 311) (Figure 1). Following removal of duplicates (149), 560 articles were considered for preliminary title and abstract screening. All original research describing mechanistic models between mammalian wildlife and livestock were included.

Preliminary review resulted in the exclusion of 501 articles. These articles only considered a single species, did not include interaction between livestock and mammalian (i.e., non-avian) wildlife, were an exclusively within-host study (i.e., molecular, microbiological, immunological, or genomic model), were of phylogenetic or phylodynamic models, used purely statistical, economic or decision-analysis models, were a review or editorial, or were experiments or field studies that did not include mechanistic modeling.

Of the 59 articles that qualified for full-text review, four articles were excluded following full-text assessment for not modeling transmission at the livestock-wildlife interface (11–14). All 10 calibration articles were captured in the search query (15–24). One article not identified in the initial search but previously known to the authors was subsequently included (25), yielding 56 articles for data extraction. Author, date, domestic and wildlife species, disease, location, domestic and wildlife model frameworks, means of domestic and wildlife representation, source of model calibration, main driver of transmission between species, interaction process between species, direction of transmission, primary research objective and main hurdles challenges or limitations were extracted.

Model frameworks were classified either by the author classification or, if not specified, the classification that best approximated the described model. Individual-based models (IBMs)—synonymous with agent-based models but chosen for its nomenclature preference in ecology—were those where populations are simulated through the complex interactions of individuals with distinct properties (26, 27). Whether individual animals or herds, in these spatially-explicit models each individual unit interacts with its environment. Conversely, population-based models—commonly referred to as compartmental models—reflected the dynamics at a population scale without accounting for individual heterogeneity. Geographic automata were a generalization of the cellular automata structure, relying on the same principles of local grid-based neighbor interactions, but no longer constraining animal populations to a uniformly-spaced lattice (28, 29). Metapopulation models were defined as those models that connect multiple subpopulations, where in the simplest form infectives in one patch can simply transmit disease to susceptibles in either their or another patch (30). Lastly, models were classified as network models when the framework relied on individual or herd connectivity through explicit networks. Of note, no standard methodology for describing individual-based epidemic models exists, which has led to irregularities and inconsistencies among model descriptions (31). Though protocols have been proposed for describing model structures in a standardized way, they are not specific to disease modeling nor are they consistently followed (31, 32).

## Results

### Epidemiological characteristics

**Figure 2:**
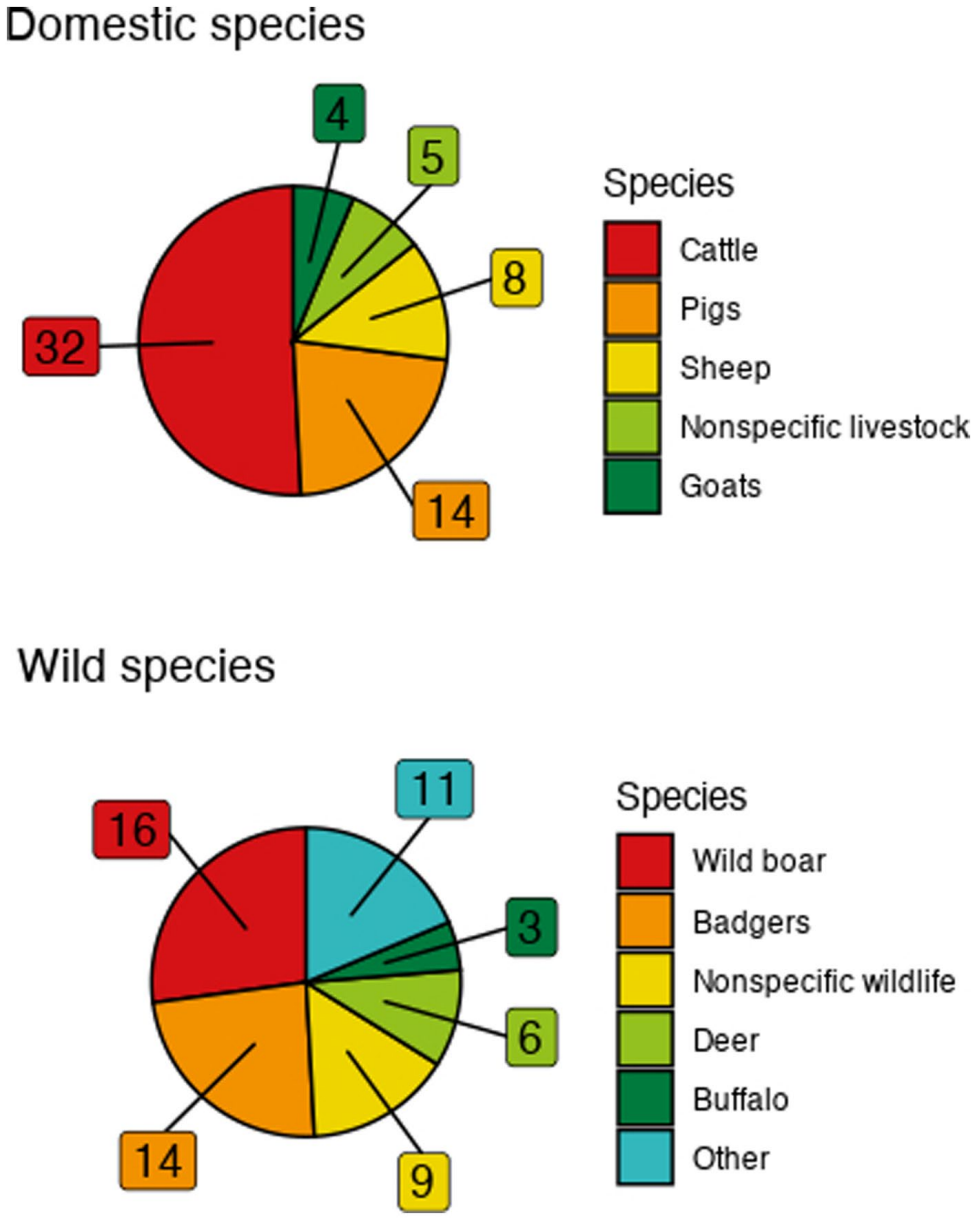
Frequency of represented species in the included models. “Other” category includes singularly-represented species consisting of bharal, bighorn sheep, bison, feral cats, feral pigs, impala, possums, Saiga antelopes, stray dogs, wildebeest, and zebra.

Publication dates ranged from 2001 to 2023 (Supplementary Table S1). Cattle were the predominant domestic species represented—being included in 32 models—followed by pigs (14), sheep (9), nonspecific livestock (5), and goats (4) (Figure 2). Combinations of livestock species (cattle, goats, pigs and sheep or cattle, goats, and sheep, or cattle and sheep, or goats and sheep) were present in four models. Among explicitly modeled wildlife, wild boar (16), badgers (13), nonspecific wildlife (9), deer (6), and buffalo (3) were most commonly represented with one model including both wild boar and deer (Figure 2). Additional wildlife was represented only once each: bharal, bighorn sheep, bison, feral cats, feral pigs, impala, possums, Saiga antelopes, stray dogs, wildebeest, and zebra.

**Figure 3:**
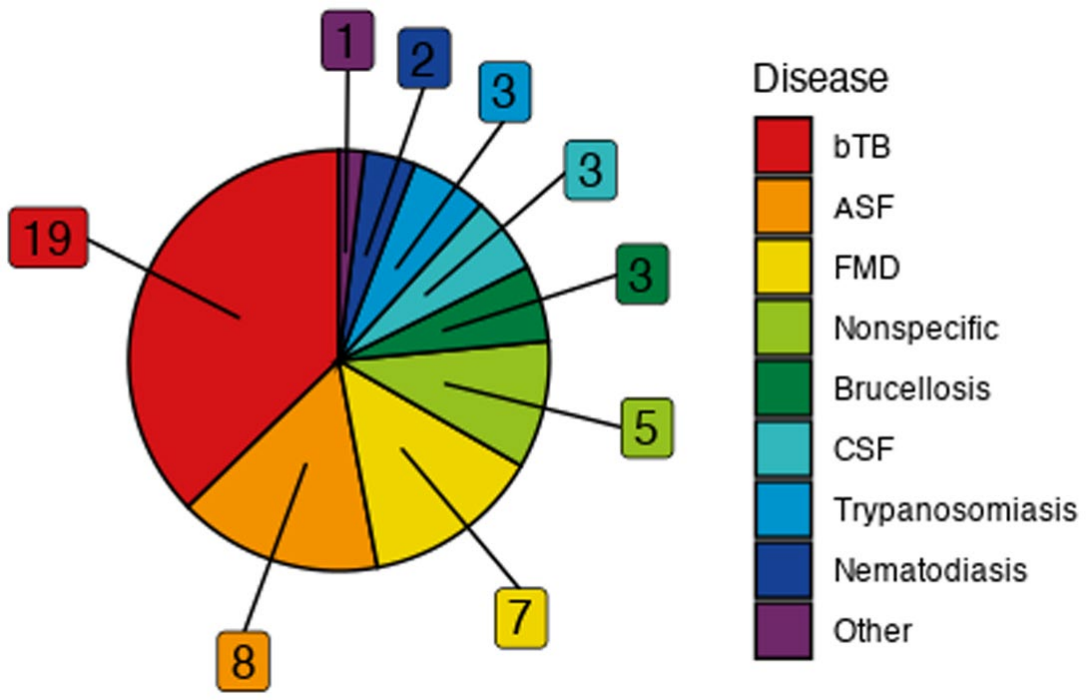
Frequency of represented diseases in the included models. “Other” category includes singularly-represented diseases of babesiosis, echinococcosis, louping ill, neosporosis, toxoplasmosis, trichostrongylosis, and paratuberculosis. Abbreviations: African swine fever (ASF), bovine tuberculosis (bTB), classical swine fever (CSF), foot and mouth disease (FMD).

Viral, bacterial, and parasitic diseases were represented among the models (Figure 3). Bovine tuberculosis (bTB) was the most frequently modeled disease (19) followed by African swine fever (ASF) (8), foot-and-mouth disease (FMD) (7), brucellosis (3), classical swine fever (CSF) (3), trypanosomiasis (3), and nematodiasis (2) (Supplementary Table S1). Babesiosis, echinococcosis, louping ill, neosporosis, toxoplasmosis, trichostrongylosis, and paratuberculosis were each represented a single time (Supplementary Table S1). Of the locations explicitly modeled, the United Kingdom (UK) and United States of America (USA) were represented the most frequently (13 and 10, respectively), and a total of 19 unique countries or regions across Africa, Europe, North America, and Oceania were represented among the studies (Supplementary Table S1). One set of studies occurred on a fictitious island for the purposes of the ASF modeling Challenge (3) (33), and nine models were not of a specific location.

### Model objectives, frameworks, and representation of hosts

The majority of primary objectives were to assess control strategies in a multihost population (26), estimate transmission risk to livestock from wildlife (9), or explain observed transmission dynamics while considering the effects of multiple hosts (8), though estimating transmission parameters (4), determining consequences of hypothetical outbreak scenarios (4), nowcasting of multihost epidemics (3), and comparing the impact of model assumptions on transmission in a multihost environment (1) were also represented (Supplementary Table S2). Models that assessed control strategies were mostly concerned with the outcomes of control strategies on livestock, whether the strategy was applied to wild hosts (10) (16, 20, 22, 34–40) or wild and domestic hosts (5) (23, 41–44). These studies were heavily focused on bTB (11) in the UK (7), ascertaining the outcomes of control strategies applied to badgers on either cattle (4) or cattle and badgers (5). Other studies examined the outcomes on domestic hosts of interventions applied to domestic hosts while accounting for transmission from wildlife, as for babesiosis, louping-ill, nematodiasis, and CSF (45–48).

Five model frameworks were used among domestic or wildlife species in the included articles: individual-based models (IBMs), population-based models (PBMs), cellular or geographic automata (CA), metapopulation, and network models. Individual-based models were the most popular framework for domestic hosts (25), whereas population-based models were the most prominent framework for wildlife species (25). The majority of models used the same frameworks for both the domestic and wildlife populations, though five articles used different model frameworks for each species. Here, network models for domestic species were used in combination with a wildlife metapopulation model (49) or PBM (50), or domestic IBMs were used with a wildlife metapopulation model (51) or wildlife PBMs (39, 52).

Among individual-based frameworks, a variety of approaches were taken to represent hosts, with point locations of farms, herds or production sites being the most common epidemiological unit for both domestic species (9) and raster cells being the most used method for wildlife (17). Indeed, representing domestic species by point locations and wildlife through a raster was the most common model combination seen in the included articles (7). Among wildlife, raster cells were predominantly based on habitat (10), though home ranges (43), host density (21, 53), and contiguous social groups (23, 41, 42, 44) were also used to define them. The models in ten articles represented species through mobile agents across a simulated landscape, with five of them using individual mobile agents for both domestic and wildlife models. In these models, mobile agents were programmed to roam over home-range polygons (54), a habitat raster (17, 45), or a lattice of cells without habitat characteristics (20, 55). Alternatively, five studies provided movement attributes to only one host, with four studies representing domestic hosts through raster cells or polygons while wildlife were represented *via* mobile agents (22, 34, 35, 40).

Population-based frameworks were used for a variety of diseases, including ASF, brucellosis, bTB, CSF, FMD, louping ill, and multiple parasitic diseases (echinococcosis, nematodiasis, neosporosis, toxoplasmosis, trichostrongylosis, and trypanosomiasis). These models represented domestic and wildlife species through parameters quantifying host abundance, though population density (16, 47, 56, 57), host presence (58), and recruitment rate (59) were also used.

Three studies used metapopulation approaches to represent wildlife, two of which were designed explicitly for the ASF Modeling Challenge (49, 60). Here, wildlife was represented through a habitat raster (49), home range polygon (51), or host-presence patches (60), while domestic species were represented through network models, metapopulation models, or IBMs using farms as location-specific network nodes (49, 60) or polygons of herd locations (51), respectively.

Cellular automata models—or their complexification to geographic automata models—were used to model FMD in Australia and the USA (19, 29, 61). A density distribution over a cellular lattice (19, 29) or a raster of herds (61) was used to represent domestic species, while wildlife species were represented *via* seasonal habitat or land cover density over a cellular lattice (19, 29) or habitat raster (61).

Network models were used to simulate ASF (49), bTB (50, 62) and brucellosis (63) transmission. Network nodes were used to represent farms, pastures, or herd types of domestic hosts and home ranges or statistic reservoirs of wildlife hosts.

### Drivers of disease transmission, representations of host interaction processes, and model calibration

Disease transmission was predominately driven through the overlap of livestock and wildlife habitat, home range, or shared pasture (18) (Supplementary Table S3). In some cases, modeled transmission was driven through wildlife escaping their home range and contacting livestock (64) or from wildlife explicitly seeking food and water sources (17). When explicit overlap was not considered, the proximity of livestock to wildlife areas or cases was used, as seen in models of ASF and CSF (24, 48, 60, 65, 66). Livestock proximity to forests (15) or livestock adjacency to hunting areas (35) was also used to drive transmission. Population-based models, frequently of parasitic disease, relied on host abundance or density to drive transmission between species (Supplementary Table S2). Wildlife dispersal in response to applied control strategies was also seen to drive transmission between species, as modeled in Byrom et al. (34) and Lintott et al. (67).

The models in this review examined transmission in all directions, with unidirectional transmission from wildlife to livestock (26) or bidirectional transmission between wildlife and livestock (25) being most frequent (Supplementary Table S3). Two models examined unidirectional disease transmission from livestock to wildlife (22, 51), and three models looked at transmission of disease between both wildlife and livestock to humans (36, 37, 63).

Different functional representations of the interaction processes between the different host populations were seen throughout the models in the included studies. Transmission rates, corresponding to the average number of new infections produced by one infectious unit per unit of time, are widely used in the literature for all modeling paradigms. This key parameter in epidemiology governs the force of infection, which might reflect either direct transmission between host or indirect transmission through vectors or environment. The transmission rate can also be defined as the product of the contact rate and the transmission probability whenever a contact occurs with an infectious unit. A few studies disentangled these two parameters to evaluate the relative impact of external factors on the different mechanisms of transmission (15, 29, 53, 61). When transmission rates were not used to represent host interaction, if the data was available, transmission or contact probability, or contact rate were also used to represent the host interaction process.

Of the 56 included studies, 37 models were calibrated *via* published literature. Only 10 of the models were calibrated to a real epidemic, and seven of those were specific to bTB (16, 18, 50, 55, 58, 62, 68). The other two real epidemics modeled were ASF in the Republic of Korea (24) and CSF in Japan (48, 65). Three more articles did model ASF, but as part of the ASF challenge and with synthetic data (49, 60, 66). Four studies included a field component that was used in model calibration (25, 34, 57, 69).

### Main hurdles

While each model had its own limitations unique to the specific scenario for which it was designed, four main classes of hurdles were identified: Lack of empirical parameter estimates, limited wildlife location data, defining what constitutes livestock-wildlife contact, and balancing model complexity with utility (Supplementary Table S3). By far, a lack of empirical parameter estimates was the primary limitation in 31 studies. The lack of empirical parameter estimates needed for model calibration could be further divided between parameters for disease transmission (17), wildlife behavior (8), livestock-wildlife contact (2), wildlife prevalence (2), interspecies control strategy effects (1), and host management (1). Even when an explicit interhost transmission study was conducted, its occurrence under controlled laboratory conditions limits extrapolation of these parameters to natural conditions (25). Limited data on wildlife locations was the primary limitation in 14 models. Here, a lack of wildlife density and/or distribution data (11), lack of environmental reservoir locations (2), or uncertainties regarding wildlife habitat use as a function of preference versus availability (1) were identified. Indeed, even with fine-grain wildlife habitat data, understanding if such habitat is preferred or simply available limits the generalizability of a model (45).

Beyond a lack of parameters for quantifying livestock-wildlife contact, even defining what constitutes an epidemiological relevant contact was the primary hurdle of 3 models (29, 64, 69). Balancing model complexity with utility was the main hurdle in 4 models. Among multihost models of vector-borne disease, incorporating explicit vector population dynamics was the primary limitation even when parameterization data was available, due to its effects on model complexity and generalizability (36, 37, 68). Conversely, one of the ASF challenge models—where all data was synthetic—was more limited by the trade-off between model complexity and computational time required for real-time modeling than any explicit wildlife parameter gaps (60).

## Discussion

Modeling disease transmission between wild and domestic species is a complex task that has been achieved through a multitude of methods but for only a few disease scenarios. Indeed, of the 118 diseases at the wildlife-livestock interface represented in the literature body, only 14 have been explored through mathematical modeling (70). From selecting model frameworks and host representations to determining the drivers of transmission that are to be included in the models, distinct populations—often with drastically differing population dynamics—must be accurately represented. These choices of methodology are a reflection of the skill set of the researcher team, the research question being addressed by the model and the availability of data. Domestic species were defined through explicit herd locations, and further delineated by additional parameters of herd density, defined pasture area, habitat and abundance. In contrast, wildlife species were often modeled through variables of habitat potential, density distribution, or population abundance. Only in a few models of badgers were the exact burrow locations known, but even then only the underground dens were identified and surrounding home ranges still had to be inferred (23, 41, 42). In choosing a paradigm to represent a system one must consider the trade-offs between complexity, comprehensibility, and underlying assumptions. Though a model should be a realistic representation, deciding on the degree of realism required—and keeping in mind that models are only synthetic representations of a phenomenon—is part of the art of model selection. The parsimony principle should always be kept in mind, especially in situations involving wildlife where parameters are difficult to document, require a high level of data for inference, or are highly variable, due to the influence of the interaction of multiple factors.

Habitability is often used as a proxy to represent wild host populations, as was the case in 11 models (15, 19, 22, 35, 40, 45, 49, 61, 65, 66, 71). Defining such suitability can involve the incorporation of landcover maps, abundance data from hunting records, expert opinion, and previously-published species distribution models. In the context of models examined in this review, species distribution is a means to the end for representing disease transmission through multiple populations, and simplifications of a species’ true distribution—especially wildlife—are evident in all livestock-wildlife disease transmission models. Combined with data limitations among wildlife species, this invariably results in wildlife disease transmission models that contain more uncertainties than those of domestic animal species (4). Indeed, monitoring infectious diseases in wild populations is far more demanding in terms of resources and time required than for livestock. Though there are innate assumptions that may not be fully accurate when using habitat suitability to represent wildlife presence, for the given modeling objectives these assumptions are acceptable. While sensitivity analyses within the selected articles focused on model parameters (e.g., transmission detection and contact rates, mortality, and initial infection location) and not the representation of the distribution of wildlife, Birch et al. (50) did assess the sensitivity of their model to the number of environmental reservoirs—identifying that that parameter was more constrained than that of the environment-to-livestock transmission rate.

By far the most-represented transmission driver was that of overlapping habitat. Whether livestock were modeled as discrete farms or mobile herds, most disease transmission was driven by locations that intersected with wildlife habitats or home ranges. Agricultural intensification, wildlife habitat fragmentation and encroachment on wild animal habitats are known global drivers of disease emergence, and these drivers are reflected in these models (72, 73). Local drivers, such as water and food-seeking behaviors of wildlife, pasture sharing between livestock and native wildlife, and outdoor husbandry, were also reflected among the models (73). Control strategies themselves can also be implicit in driving transmission, as culling can have an opposite-as-intended effect increasing both disease prevalence and number of infectious individuals (74, 75). This was reflected in models of bTB transmission as when Smith et al. (43) used a perturbation parameter to account for the increase in transmission from culling, or studied by Lintott et al. (67) to quantify the impact of dispersal following disease control.

Included studies that focused on control strategy assessments invariably quantified the number of infected herds, as explicitly stated in Pineda-Krch et al. (53), Ramsey et al. (40), and Smith et al. (41, 43), but certain methodologies precluded the ability to determine the relative contribution of species to overall spread. For instance, when foot-and-mouth disease was investigated among feral pigs and livestock, a single-layer cellular automata model was used (19). Therefore, multiple species had to be mutated into a composite herd that varied based on a species-specific infectivity parameter (depending on the type and number of each specie). Though effective at discerning the overall epizootic spatio-temporal pattern, such a method did not allow for the disentangling of individual species’ contribution. Of the models that tried to explain observed transmission dynamics, the relative contribution of involved species to epizootic propagation was only ascertained in a few models (18, 24, 50, 58). Indeed, mechanistic models that are based on specific spatio-temporal case data and uncertain population distributions (particularly for wildlife), and for which inter-species transmission events are not directly observable, may be very challenging to estimate relative contributions. More direct evidence of disease spill-over, as can be obtained through genomic approaches based on pathogen sequences, could be a useful complement to further inform such models (76).

The challenges of multispecies modeling have been extensively reviewed in the literature. Whether focused on the human-wildlife (2) or the domestic-wildlife (3, 6) interface, or more broadly examining modeling of multihost systems (1, 77), all reviews espouse that though hurdles have been overcome, many more challenges remain in need of address. Huyvaert et al. (3) identified that these challenges fall into three broad categories relating to host and pathogen distribution and movement, transmission pathways and rates, and the effects of disease and mitigation on host populations. Five years later, these hurdles continue to be represented in the 14 included studies published since 2018. Investments in ecological research with project planning input from ecological modelers, infectious disease specialists (including epidemiologists, veterinarians, and virologists) and wildlife managers—among many additional critical fields at this interface—may help to overcome these challenges, through enabling the studies needed to elucidate the parameters needed for modeling this interface.

The need for additional modeling at the livestock-wildlife interface is supported by the ever-increasing interactions between wildlife and livestock. Livestock production systems constitute the largest use of land in the world, and increasing global food demand invariably results in the expansion of these systems (73). The consequent deforestation that makes room for these enterprises results in the juxtaposition of livestock with wildlife, increasing the areas of interaction between the two (72, 73). Climate change has had profound effects at both global and local scales. Large-scale shifts in vector distributions have resulted in outbreaks of diseases that were formerly confined to tropical regions, as seen with bluetongue virus (73, 78). Locally, water scarcity in arid and semi-arid regions has resulted in mixed congregations around available water sources for pastoral livestock and wildlife (73).

In the majority of rural communities, backyard farming and small-scale animal production systems constitute the primary livelihoods and food sources (79). These low-biosecurity operations permit regular contact between livestock and wildlife, and have often been central to outbreaks of diseases shared at this interface—including ASF, CSF, FMD, brucellosis, and rabies (73, 80). Improved animal welfare in high-income countries has also resulted in increases in the number of outdoor and open-air production systems, which also puts livestock at higher risk of wildlife contacts (73). The livestock-wildlife interface acts as an important area of infectious disease propagation, and mathematical models are able to investigate and quantify the involved dynamics, helping to improve our understanding of these drivers of transmission and contribute to the conception of holistic control strategies.

## Data availability statement

The original contributions presented in the study are included in the article/Supplementary material, further inquiries can be directed to the corresponding author.

## Author contributions

BH, TV, MA, and NR contributed to conception and design of the study. BH performed the data extraction, analysis, and composed the first draft of the manuscript. All authors contributed to manuscript revision, read, and approved the submitted version.

## Conflict of interest

The authors declare that the research was conducted in the absence of any commercial or financial relationships that could be construed as a potential conflict of interest.

## Publisher’s note

All claims expressed in this article are solely those of the authors and do not necessarily represent those of their affiliated organizations, or those of the publisher, the editors and the reviewers. Any product that may be evaluated in this article, or claim that may be made by its manufacturer, is not guaranteed or endorsed by the publisher.
