# Supplementary material for: Mathematical modeling at the livestock-wildlife interface: scoping review of drivers of disease transmission between species
Source: Front Vet Sci. 2023 Sep 6;10:1225446. doi: 10.3389/fvets.2023.1225446 (PMC10511766; doi:10.3389/fvets.2023.1225446)
Supplement: Supplementary file 1 [file Table_1.docx]

Supplementary Material

Mathematical modelling at the livestock-wildlife interface: Scoping review of drivers of disease transmission between species

Brandon H Hayes^*1,2^, Timothée Vergne^1^, Mathieu Andraud^2^, Nicolas Rose^2^

^1^ IHAP, Université de Toulouse, INRAE, ENVT, Toulouse, France

^2^ Ploufragan-Plouzané-Niort Laboratory, the French Agency for Food, Agriculture and the Environment (ANSES), Ploufragan, France

# * Correspondence: Brandon H Hayes [brandon.hayes@envt.fr](mailto:brandon.hayes@envt.fr)

# Supplementary Tables 1-3

Table S1. Epidemiological characteristics of included articles.

| Reference | Domestic species | Wildlife species | Disease | Location |
| --- | --- | --- | --- | --- |
| Agudelo et al., 2021 (45) | Cattle | Deer | Babesiosis | USA |
| Beaunee et al., 2023 (49) | Pigs | Wild boar | ASF | Fictitious island |
| Birch et al., 2018 (50) | Cattle | Badgers | bTB | UK |
| Boklund et al., 2008 (15) | Pigs | Wild boar | CSF | Denmark |
| Bouchez-Zacria et al., 2018 (62) | Cattle | Badgers | bTB | France |
| Brooks-Pollock et al., 2015 (58) | Cattle | Badgers | bTB | UK |
| Byrom et al., 2015 (34) | Nonspecific livestock | Possum | bTB | New Zealand |
| Carpenter et al., 2014 (51) | Sheep | Bighorn sheep | Nonspecific | USA |
| Cosgrove et al., 2018 (35) | Cattle | Deer | bTB | USA |
| Cox et al., 2005 (16) | Cattle | Badgers | bTB | UK |
| Dankwa et al., 2022 (60) | Pigs | Wild boar | ASF | Fictitious island |
| Dion et al., 2011 (17) | Cattle | Buffalo | FMD | South Africa |
| Donnelly et al., 2013 (18) | Cattle | Badgers | bTB | UK |
| Doran et al., 2005 (19) | Cattle, sheep | Wild boar | FMD | Australia |
| Greenman and Hoyle, 2008 (56) | Nonspecific livestock | Nonspecific wildlife | bTB | Nonspecific |
| Hargrove et al., 2012 (36) | Cattle | Nonspecific wildlife | Trypanosomiasis | Uganda |
| Hayama et al., 2020 (65) | Pigs | Wild boar | CSF | Japan |
| Jori and Etter, 2016 (64) | Cattle | Buffalo | FMD | South Africa |
| Kajunguri et al., 2014 (37) | Cattle | Nonspecific wildlife | Trypanosomiasis | Nonspecific |
| Khanyari et al., 2021 (57) | Goats, sheep | Bharal | Nematodiasis | India |
| Kilpatrick et al., 2009 (81) | Cattle | Bison | Brucellosis | USA |
| Laffan et al., 2011 (29) | Cattle | Wild boar | FMD | USA |
| Lintott et al., 2013 (67) | Nonspecific livestock | Nonspecific wildlife | Nonspecific | Nonspecific |
| Manlove et al., 2019 (20) | Nonspecific livestock | Nonspecific wildlife | Nonspecific | Nonspecific |
| Marion et al., 2008 (82) | Cattle | Nonspecific wildlife | Nonspecific | Nonspecific |
| Mateus-Pinilla et al., 2002 (38) | Pigs | Cats | Toxoplasmosis | Nonspecific |
| Moreno-Torres et al., 2017 (69) | Cattle | Deer | Neosporosis | USA |
| Morgan et al., 2007 (83) | Sheep | Saiga antelopes | Trichostrongylosis | Kazakhstan |
| Moustakas and Evans, 2015 (55) | Cattle | Badgers | bTB | UK |
| Mugabi and Duffy, 2023 (59) | Pigs | Wild boar | ASF | East and Southern Africa |
| Muñoz et al., 2022 (66) | Pigs | Wild boar | ASF | Fictitious island |
| Mur et al., 2018 (21) | Pigs | Wild boar | ASF | Sardinia |
| Nyerere et al., 2020 (84) | Nonspecific livestock | Nonspecific wildlife | Brucellosis | Nonspecific |
| Odeniran et al., 2020 (39) | Cattle, goats, sheep | Nonspecific wildlife | Trypanosomiasis | West Africa |
| O'Hare et al., 2014 (68) | Cattle | Badgers | bTB | UK |
| Patterson et al., 2022 (71) | Pigs | Feral pigs | Nonspecific | USA |
| Phepa et al., 2016 (85) | Cattle | Buffalo | bTB | South Africa |
| Pietschmann et al., 2015 (25) | Pigs | Wild boar | ASF | Nonspecific |
| Pineda-Krch et al., 2010 (53) | Cattle, goats, pigs, sheep | Wild boar | FMD | USA |
| Porter et al., 2011 (46) | Sheep | Deer | Louping ill | UK |
| Ramsey et al., 2016 (40) | Cattle | Deer | bTB | USA |
| Rong et al., 2021 (86) | Sheep | Stray dogs | Echinococcosis | China |
| Roy et al., 2011 (63) | Cattle | Nonspecific wildlife | Brucellosis | Sub-Saharan Africa |
| Smith et al., 2001a (41) | Cattle | Badgers | bTB | UK |
| Smith et al., 2001b (42) | Cattle | Badgers | bTB | UK |
| Smith et al., 2009 (52) | Cattle | Badgers | bTB, paratuberculosis | Nonspecific |
| Smith et al., 2012 (44) | Cattle | Badgers | bTB | UK |
| Smith et al., 2016 (43) | Cattle | Badgers | bTB | UK |
| Taylor et al., 2021 (22) | Pigs | Wild boar | ASF | Europe |
| Walker et al., 2018 (47) | Goats | Impala, zebra, wildebeest | Nematodiasis | Bostwana |
| Ward et al., 2011 (61) | Cattle | Wild boar, deer | FMD | USA |
| Ward et al., 2015 (54) | Cattle | Wild boar | FMD | Australia |
| Wilkinson et al., 2004 (23) | Cattle | Badgers | bTB | UK |
| Wilkinson et al., 2009 (87) | Cattle | Badgers | bTB | UK |
| Yang and Nishiura, 2022 (48) | Pigs | Wild boar | CSF | Japan |
| Yoo et al., 2021 (24) | Pigs | Wild boar | ASF | Republic of Korea |

Table S2. Model objectives, frameworks, and means of species representation.

| Reference | Primary research objective | Domestic model framework | Domestic representation | Wildlife model framework | Wildlife representation |
| --- | --- | --- | --- | --- | --- |
| Agudelo et al., 2021 (45) | Assess control strategies | IBM | Mobile agents over raster cells (habitat) | IBM | Mobile agents over raster cells (habitat) |
| Beaunee et al., 2023 (49) | Epidemic nowcasting | Network | Network nodes (farms) | Metapopulation | Raster cells (habitat) |
| Birch et al., 2018 (50) | Explain observed transmission dynamics | Network | Network nodes (farms) | PBM | Parameter (farm-specific FoI) |
| Boklund et al., 2008 (15) | Estimate transmission risk | IBM | Point locations (herds) | IBM | Point array (from habitat raster) |
| Bouchez-Zacria et al., 2018 (62) | Explain observed transmission dynamics | Network | Network nodes (pastures) | Network | Network nodes (home ranges) |
| Brooks-Pollock et al., 2015 (58) | Explain observed transmission dynamics | PBM | Parameter (host presence) | PBM | Parameter (host presence) |
| Byrom et al., 2015 (34) | Assess control strategies | IBM | Polygons (pasture) | IBM | Mobile agents over polygon |
| Carpenter et al., 2014 (51) | Determine consequences of hypothetical outbreaks | IBM | Polygons (herd locations) | Metapopulation | Home range polygons |
| Cosgrove et al., 2018 (35) | Assess control strategies | IBM | Raster cells (farm density) | IBM | Mobile agents over raster cells (habitat) |
| Cox et al., 2005 (16) | Assess control strategies | PBM | Parameter (host density) | PBM | Parameter (host density) |
| Dankwa et al., 2022 (60) | Epidemic nowcasting | Metapopulation | Network nodes (farms) | Metapopulation | Patches |
| Dion et al., 2011 (17) | Estimate transmission risk | IBM | Mobile agents over raster cells (habitat) | IBM | Mobile agents over raster cells (habitat) |
| Donnelly et al., 2013 (18) | Explain observed transmission dynamics | PBM | Parameter (host abundance) | PBM | Parameter (host abundance) |
| Doran et al., 2005 (19) | Determine consequences of hypothetical outbreaks | Cellular automata | Cellular lattice (density distribution) | Cellular automata | Cellular lattice (density and habitat) |
| Greenman and Hoyle, 2008 (56) | Assess control strategies | PBM | Parameter (host density) | PBM | Parameter (host density) |
| Hargrove et al., 2012 (36) | Assess control strategies | PBM | Parameter (host abundance) | PBM | Parameter (host abundance) |
| Hayama et al., 2020 (65) | Estimate transmission risk | IBM | Point locations (farms) | IBM | Raster cells (habitat) |
| Jori and Etter, 2016 (64) | Estimate transmission risk | PBM | Parameter (host abundance) | PBM | Parameter (host abundance) |
| Kajunguri et al., 2014 (37) | Assess control strategies | PBM | Parameter (host abundance) | PBM | Parameter (host abundance) |
| Khanyari et al., 2021 (57) | Assess control strategies | PBM | Parameter (host density) | PBM | Parameter (host density) |
| Kilpatrick et al., 2009 (81) | Estimate transmission risk | PBM | Parameter (host abundance) | PBM | Parameter (host abundance) |
| Laffan et al., 2011 (29) | Compare impact of model assumptions | Geographic automata | Cellular lattice (density distribution) | Geographic automata | Cellular lattice (land cover) |
| Lintott et al., 2013 (67) | Assess control strategies | PBM | Parameter (host abundance) | PBM | Parameter (host abundance) |
| Manlove et al., 2019 (20) | Assess control strategies | IBM | Mobile agents over lattice | IBM | Mobile agents over lattice |
| Marion et al., 2008 (82) | Estimate transmission risk | IBM | Patch lattice | IBM | Parameter (host presence) |
| Mateus-Pinilla et al., 2002 (38) | Assess control strategies | PBM | Parameter (host abundance) | PBM | Parameter (host abundance) |
| Moreno-Torres et al., 2017 (69) | Estimate parameters (reproductive number) | PBM | Parameter (host abundance) | PBM | Parameter (host abundance) |
| Morgan et al., 2007 (83) | Explain observed transmission dynamics | PBM | Parameter (host abundance) | PBM | Parameter (host abundance) |
| Moustakas and Evans, 2015 (55) | Assess control strategies | IBM | Mobile agents over lattice | IBM | Mobile agents over lattice |
| Mugabi and Duffy, 2023 (59) | Explain observed transmission dynamics | PBM | Parameter (recruitment rate) | PBM | Parameter (recruitment rate) |
| Muñoz et al., 2022 (66) | Epidemic nowcasting | IBM | Point locations (production sites) | IBM | Raster cells (habitat) |
| Mur et al., 2018 (21) | Estimate transmission risk | IBM | Point locations (farms) | IBM | Raster cells (host density) |
| Nyerere et al., 2020 (84) | Explain observed transmission dynamics | PBM | Parameter (host abundance) | PBM | Parameter (host abundance) |
| Odeniran et al., 2020 (39) | Assess control strategies | IBM | Parameter (host abundance) | PBM | Parameter (host abundance) |
| O'Hare et al., 2014 (68) | Estimate parameters (reproductive number) | PBM | Parameter (host abundance) | PBM | Parameter (host abundance) |
| Patterson et al., 2022 (71) | Estimate transmission risk | IBM | Point locations (farms) | IBM | Raster cells (habitat) |
| Phepa et al., 2016 (85) | Estimate parameters (reproductive number) | PBM | Parameter (host abundance) | PBM | Parameter (host abundance) |
| Pietschmann et al., 2015 (25) | Estimate parameters (reproductive number) | PBM | Parameter (host abundance) | PBM | Parameter (host abundance) |
| Pineda-Krch et al., 2010 (53) | Determine consequences of hypothetical outbreaks | IBM | Point locations (herds) | IBM | Raster cells (host density) |
| Porter et al., 2011 (46) | Assess control strategies | PBM | Parameter (host abundance) | PBM | Parameter (host abundance) |
| Ramsey et al., 2016 (40) | Assess control strategies | IBM | Raster cells (farm density) | IBM | Mobile agents over raster cells (habitat) |
| Rong et al., 2021 (86) | Assess control strategies | PBM | Parameter (host abundance) | PBM | Parameter (host abundance) |
| Roy et al., 2011 (63) | Assess control strategies | Network | Network nodes (herd type) | Network | Network nodes (static reservoir) |
| Smith et al., 2001a (41) | Assess control strategies | IBM | Point locations (herds) | IBM | Raster cells (contiguous social groups) |
| Smith et al., 2001b (42) | Assess control strategies | IBM | Point locations (herds) | IBM | Raster cells (contiguous social groups) |
| Smith et al., 2009 (52) | Estimate transmission risk | IBM | Mobile agents | PBM | Parameter (fecal contamination) |
| Smith et al., 2012 (44) | Assess control strategies | IBM | Polygons (pasture) | IBM | Raster cells (contiguous social groups) |
| Smith et al., 2016 (43) | Assess control strategies | IBM | Polygons (pasture) | IBM | Raster cells (home ranges) |
| Taylor et al., 2021 (22) | Assess control strategies | IBM | Raster cells (herd density) | IBM | Mobile agents over raster cells (habitat) |
| Walker et al., 2018 (47) | Assess control strategies | PBM | Parameter (host density) | PBM | Parameter (host density) |
| Ward et al., 2011 (61) | Determine consequences of hypothetical outbreaks | Geographic automata | Cellular lattice (density distribution) | Geographic automata | Cellular lattice (land cover) |
| Ward et al., 2015 (54) | Assess control strategies | IBM | Mobile agents over home range polygons | IBM | Mobile agents over home range polygons |
| Wilkinson et al., 2004 (23) | Assess control strategies | IBM | Raster cells (herds) | IBM | Raster cells (contiguous social groups) |
| Wilkinson et al., 2009 (87) | Assess control strategies | IBM | Raster cells (herds) | IBM | Raster cells (contiguous social groups) |
| Yang and Nishiura, 2022 (48) | Assess control strategies | PBM | Parameter (host abundance) | PBM | Parameter (host abundance) |
| Yoo et al., 2021 (24) | Explain observed transmission dynamics | IBM | Point locations (herds) | IBM | Point locations (cases) |

Table S3. Model calibration, transmission processes, and main hurdles.

| Reference | Source of calibration | Main driver of transmission between species | Interaction process between species | Transmission direction | Main hurdles to overcome |
| --- | --- | --- | --- | --- | --- |
| Agudelo et al., 2021 (45) | Literature | Shared pasture | Contact rate | Wildlife <> livestock | Defining wildlife locations (habitat preference vs availability) |
| Beaunee et al., 2023 (49) | Simulated epidemic | Overlap of habitats or home ranges | Transmission rate | Wildlife <> livestock | Estimating parameters from real-time data |
| Birch et al., 2018 (50) | Real epidemic | Explicit contact | Transmission rate | Wildlife > livestock | Defining wildlife locations (environmental reservoirs) |
| Boklund et al., 2008 (15) | Literature | Proximity to forests | Contact rate, transmission probability | Wildlife <> livestock | Lack of empirical parameter estimates (transmission) |
| Bouchez-Zacria et al., 2018 (62) | Real epidemic | Explicit contact | Contact probability | Wildlife > livestock | Defining wildlife locations (environmental reservoirs) |
| Brooks-Pollock et al., 2015 (58) | Real epidemic | Disease prevalence in wild hosts | Transmission rate | Wildlife <> livestock | Lack of empirical parameter estimates (wildlife prevalence) |
| Byrom et al., 2015 (34) | Field experiment | Wildlife dispersal | Risk of contact | Wildlife > livestock | Lack of empirical parameter estimates (wildlife behavior) |
| Carpenter et al., 2014 (51) | Literature | Shared pasture | Transmission probability | Livestock > wildlife | Lack of empirical parameter estimates (livestock-wildlife contact) |
| Cosgrove et al., 2018 (35) | Literature | Livestock adjacency to hunting areas | Transmission rate | Wildlife > livestock | Lack of empirical parameter estimates (wildlife behavior) |
| Cox et al., 2005 (16) | Real epidemic | Disease prevalence in wild hosts | Transmission rate | Wildlife <> livestock | Defining wildlife locations (wildlife distribution & density) |
| Dankwa et al., 2022 (60) | Simulated epidemic | Livestock distance to infectious wildlife | Transmission rate | Wildlife > livestock | Balancing model complexity with utility (generalizability, speed) |
| Dion et al., 2011 (17) | Literature | Explicit contact | Risk of contact | Wildlife <> livestock | Lack of empirical parameter estimates (transmission) |
| Donnelly et al., 2013 (18) | Real epidemic | Disease prevalence in wild hosts | Transmission rate | Wildlife > livestock | Lack of empirical parameter estimates (control strategy effects) |
| Doran et al., 2005 (19) | Literature | Explicit contact | Transmission probability | Wildlife <> livestock | Defining wildlife locations (wildlife distribution & density) |
| Greenman and Hoyle, 2008 (56) | n/a | Host abundance or density | Transmission rate | Wildlife > livestock | - |
| Hargrove et al., 2012 (36) | Literature | Host abundance or density | Transmission probability | Livestock+wildlife > human | Balancing model complexity with utility (generalizability, speed) |
| Hayama et al., 2020 (65) | Real epidemic | Livestock distance to infectious wildlife | Transmission rate | Wildlife > livestock | Defining wildlife locations (wildlife distribution & density) |
| Jori and Etter, 2016 (64) | Literature | Explicit contact | Contact probability | Wildlife > livestock | Defining livestock-wildlife contact |
| Kajunguri et al., 2014 (37) | Literature | Host abundance or density | Transmission probability | Livestock+wildlife > human | Balancing model complexity with utility (generalizability, speed) |
| Khanyari et al., 2021 (57) | Field experiment | Shared pasture | Transmission rate | Wildlife <> livestock | Defining wildlife locations (wildlife distribution) |
| Kilpatrick et al., 2009 (81) | Literature | Shared pasture | Contact probability | Wildlife > livestock | Defining wildlife locations (wildlife distribution) |
| Laffan et al., 2011 (29) | Literature | Overlap of habitats or home ranges | Contact rate, transmission probability | Wildlife <> livestock | Defining livestock-wildlife contact |
| Lintott et al., 2013 (67) | Literature | Wildlife dispersal | Transmission rate | Wildlife <> livestock | Lack of empirical parameter estimates (transmission) |
| Manlove et al., 2019 (20) | n/a | Explicit contact | Contact rate | Wildlife <> livestock | Lack of empirical parameter estimates (transmission) |
| Marion et al., 2008 (82) | Literature | Shared pasture | Risk of contact | Wildlife > livestock | Lack of empirical parameter estimates (wildlife behavior) |
| Mateus-Pinilla et al., 2002 (38) | Literature | Host abundance or density | Transmission probability | Wildlife > livestock | Lack of empirical parameter estimates (wildlife prevalence) |
| Moreno-Torres et al., 2017 (69) | Field experiment | Host abundance or density | Transmission rate | Wildlife <> livestock | Defining livestock-wildlife contact |
| Morgan et al., 2007 (83) | Literature | Shared pasture | Contact rate | Wildlife <> livestock | Lack of empirical parameter estimates (wildlife behavior) |
| Moustakas and Evans, 2015 (55) | Real epidemic | Livestock housing and wildlife control strategies | Transmission rate | Wildlife <> livestock | Lack of empirical parameter estimates (transmission) |
| Mugabi and Duffy, 2023 (59) | Literature | Explicit contact | Transmission rate | Wildlife <> livestock | Lack of empirical parameter estimates (transmission) |
| Muñoz et al., 2022 (66) | Simulated epidemic | Livestock distance to infectious wildlife | Transmission probability | Wildlife > livestock | Lack of empirical parameter estimates (transmission) |
| Mur et al., 2018 (21) | Literature | Host abundance or density | Risk of contact | Wildlife > livestock | Defining wildlife locations (wildlife distribution & density) |
| Nyerere et al., 2020 (84) | Literature | Host abundance or density | Transmission rate | Wildlife <> livestock | Lack of empirical parameter estimates (transmission) |
| Odeniran et al., 2020 (39) | Literature | Host abundance or density | Transmission rate | Wildlife <> livestock | - |
| O'Hare et al., 2014 (68) | Real epidemic | Constant infectious pressure from wildlife | Transmission rate | Wildlife > livestock | Balancing model complexity with utility (generalizability, speed) |
| Patterson et al., 2022 (71) | Literature | Overlap of habitats or home ranges | Risk of contact | Wildlife > livestock | Defining wildlife locations (wildlife distribution) |
| Phepa et al., 2016 (85) | Literature | Host abundance or density | Contact rate | Wildlife <> livestock | - |
| Pietschmann et al., 2015 (25) | Field experiment | Explicit contact | Transmission rate | Wildlife <> livestock | Lack of empirical parameter estimates (transmission) |
| Pineda-Krch et al., 2010 (53) | Literature | Overlap of habitats or home ranges | Contact rate, transmission probability | Wildlife > livestock | Defining wildlife locations (wildlife distribution) |
| Porter et al., 2011 (46) | Literature | Overlap of habitats or home ranges | Transmission rate | Wildlife > livestock | Lack of empirical parameter estimates (transmission) |
| Ramsey et al., 2016 (40) | Literature | Overlap of habitats or home ranges | Transmission rate | Wildlife > livestock | Lack of empirical parameter estimates (livestock-wildlife contact) |
| Rong et al., 2021 (86) | Literature | Host abundance or density | Transmission rate | Wildlife <> livestock | Lack of empirical parameter estimates (transmission) |
| Roy et al., 2011 (63) | Literature | Constant infectious pressure from wildlife | Transmission rate | Livestock+wildlife > human | Lack of empirical parameter estimates (transmission) |
| Smith et al., 2001a (41) | Literature | Direct connection to wildlife social group | Transmission probability | Wildlife > livestock | Lack of empirical parameter estimates (wildlife behavior) |
| Smith et al., 2001b (42) | Literature | Direct connection to wildlife social group | Transmission probability | Wildlife > livestock | Lack of empirical parameter estimates (wildlife behavior) |
| Smith et al., 2009 (52) | Literature | Shared pasture | Transmission probability | Wildlife > livestock | Lack of empirical parameter estimates (wildlife behavior) |
| Smith et al., 2012 (44) | Literature | Overlap of habitats or home ranges | Transmission probability | Wildlife <> livestock | Lack of empirical parameter estimates (wildlife behavior) |
| Smith et al., 2016 (43) | Literature | Overlap of habitats or home ranges | Transmission rate | Wildlife <> livestock | Lack of empirical parameter estimates (transmission) |
| Taylor et al., 2021 (22) | Literature | Overlap of habitats or home ranges | Transmission probability | Livestock > wildlife | Defining wildlife locations (wildlife distribution & density) |
| Walker et al., 2018 (47) | Literature | Shared pasture | Transmission rate | Wildlife > livestock | Lack of empirical parameter estimates (host management) |
| Ward et al., 2011 (61) | Literature | Proximity to infectious population | Contact rate, transmission probability | Wildlife <> livestock | Lack of empirical parameter estimates (transmission) |
| Ward et al., 2015 (54) | Literature | Overlap of habitats or home ranges | Transmission probability | Wildlife <> livestock | Defining wildlife locations (wildlife distribution & density) |
| Wilkinson et al., 2004 (23) | Literature | Overlap of habitats or home ranges | Transmission probability | Wildlife > livestock | Lack of empirical parameter estimates (transmission) |
| Wilkinson et al., 2009 (87) | Literature | Overlap of habitats or home ranges | Transmission probability | Wildlife <> livestock | Lack of empirical parameter estimates (transmission) |
| Yang and Nishiura, 2022 (48) | Real epidemic | Livestock distance to infectious wildlife | Transmission probability | Wildlife > livestock | Defining wildlife locations (wildlife distribution & density) |
| Yoo et al., 2021 (24) | Real epidemic | Livestock distance to infectious wildlife | Transmission probability | Wildlife > livestock | Lack of empirical parameter estimates (transmission) |

**Additional References**

81. Kilpatrick AM, Gillin CM, Daszak P. Wildlife-livestock conflict: the risk of pathogen transmission from bison to cattle outside Yellowstone National Park. J Appl Ecol. (2009) 46:476–85. doi: 10.1111/j.1365-2664.2008.01602.x

82. Marion G, Smith LA, Swain DL, Davidson RS, Hutchings MR. Agent-based modelling of foraging behaviour: the impact of spatial heterogeneity on disease risks from faeces in grazing systems. J Agric Sci. (2008) 146:507–20. doi: 10.1017/ S0021859608008022

83. Morgan ER, Medley GF, Torgerson PR, Shaikenov BS, Milner-Gulland EJ. Parasite transmission in a migratory multiple host system. Ecol Model. (2007) 200:511–20. doi: 10.1016/j.ecolmodel.2006.09.002

84. Nyerere N, Luboobi LS, Mpeshe SC, Shirima GM. Modeling the impact of seasonal weather variations on the infectiology of brucellosis. Comput Math Methods Med. (2020) 2020:8972063. doi: 10.1155/2020/8972063

85. Phepa PB, Chirove F, Govinder KS. Modelling the role of multi-transmission routes in the epidemiology of bovine tuberculosis in cattle and buffalo populations. Math Biosci. (2016) 277:47–58. doi: 10.1016/j.mbs.2016.04.003

86. Rong X, Fan M, Zhu H, Zheng Y. Dynamic modeling and optimal control of cystic echinococcosis. Infect Dis Poverty. (2021) 10:38. doi: 10.1186/s40249-021-00807-6

87. Wilkinson D, Bennett R, McFarlane I, Rushton S, Shirley M, Smith GC. Costbenefit analysis model of badger (Meles meles) culling to reduce cattle herd tuberculosis breakdowns in Britain, with particular reference to badger perturbation. J Wildl Dis. (2009) 45:1062–88. doi: 10.7589/0090-3558-45.4.1062
